# Access to robot-assisted total knee arthroplasty varies significantly by race/ethnicity

**DOI:** 10.1186/s43019-024-00255-0

**Published:** 2025-01-06

**Authors:** Jessica Schmerler, Victoria E. Bergstein, Whitney Kagabo, Harpal S. Khanuja, Julius K. Oni, Vishal Hegde

**Affiliations:** 1 Department of Orthopaedic Surgery, The Johns Hopkins University School of Medicine, 601 N Caroline St, Baltimore, MD 21287 USA; 2 Department of Orthopaedic Surgery, The Johns Hopkins University, 4940 Eastern Avenue, Bayview Medical Offices Building, 1st Floor, Baltimore, MD 21224 USA

**Keywords:** Total knee arthroplasty, Robot-assisted total knee arthroplasty, Disparities, Race/ethnicity, Access to care

## Abstract

**Background:**

Racial/ethnic disparities in access to total knee arthroplasty (TKA) have been extensively demonstrated. Over the past several years, there has been a rapid increase in the utilization of robot-assisted TKA (RA-TKA). Therefore, this study sought to determine whether previously established racial/ethnic disparities extend to access to RA-TKA relative to conventional TKA.

**Methods:**

Patients who underwent TKA from 1 January 2022 to 31 December 2022 were identified in the National Surgical Quality Improvement Program database. Patients were stratified by whether they underwent RA-TKA. Multivariable logistic regressions, controlling for demographics and comorbidities significantly different on univariate analysis, were constructed to determine whether race/ethnicity was associated with likelihood of undergoing RA-TKA relative to conventional TKA.

**Results:**

Of the 47,898 patients who underwent TKA in 2022, 8560 (17.9%) underwent RA-TKA. On multivariable analysis, Black, Hispanic, Asian, and all other races were significantly less likely than white patients to undergo RA-TKA relative to conventional TKA (OR 0.65, 95% CI 0.59–0.70, *P* < 0.001; OR 0.70, 95% CI 0.64–0.77, *P* < 0.001; OR 0.65, 95% CI 0.55–0.76, *P* < 0.001; OR 0.78, 95% CI 0.66–0.92, *P* = 0.003, respectively).

**Conclusions:**

The results of this study demonstrate that non-white race is associated with a significantly lower likelihood of undergoing RA-TKA relative to conventional TKA. Importantly, this reduced access to RA-TKA may represent a broader disparity in access to emerging technologies and modern care. Future work should endeavor to identify drivers of this disparity to better understand minority access to emerging technologies in TKA.

*Level of evidence* III.

## Background

Total knee arthroplasty (TKA) is a common and effective procedure, providing relief for patients suffering from end-stage knee osteoarthritis [1]. There are an estimated 790,000 TKAs performed in the USA annually, and this figure is projected to grow to 3.4 million TKAs performed annually by 2040 [2, 3]. In light of this extensive volume, it is crucial to investigate factors and technologies that can improve outcomes and efficiency, especially given that an estimated 10% of patients report dissatisfaction following TKA [4]. One technology that has emerged over the past two decades has been robot-assisted TKA (RA-TKA), which aids surgeons in precision of bone cutting, mechanical alignment restoration, and implant positioning [5]. Although whether RA-TKA improves outcomes remains controversial [6, 7], factors such as patient marketing, vendor promotion, and increased accuracy during cases have led to rapid adoption of RA-TKA in recent years. Studies show a greater than 600% increase in utilization over 2015–2020, and utilization doubling again over 2020–2022 to account for nearly 15% of all TKAs performed in the USA [8, 9].

Despite the success and efficacy of TKA, there is extensive literature reporting racial disparities associated with the procedure [10–13]. There is a demonstrated lower rate of TKA utilization in Black, Hispanic, Asian, Native American, and mixed race patients compared with white patients, but this discrepancy is not explained by decreased disease prevalence or disability in these minority populations [10, 14]. Disparities have also been seen in postoperative TKA outcomes (including higher rates of complications, readmissions, revision, and mortality) and lower margins of improvement in patient-reported outcome measures (PROMs) [15]. Predisposing factors to these racial disparities in TKA include differing health literacy, provider bias, lower rates of health insurance coverage, higher utilization of lower quality hospitals, and systemic racism, among others [15]. When considering RA-TKA in particular, studies of time periods beginning in 2012 and 2016 have found lower rates of RA-TKA in Black patients compared with white patients [16, 17]. With the aforementioned more recent rapidly increasing adoption of RA-TKA since 2020 [9], it remains important to continue to investigate racial/ethnic disparities in access to this emerging technology. Furthermore, regardless of the impact of RA-TKA on outcomes, establishing the existence of disparities in access to emerging technologies encourages policies aimed at reducing them. This, in turn, may decrease disparities in access to future technologies that may end up being beneficial to patients.

Given the relative paucity of literature studying racial disparities regarding RA-TKA access, the purpose of this study was to evaluate how the utilization of RA-TKA compared with conventional TKA varies by patient race/ethnicity. Considering the abundance of previous literature reporting on racial disparities that exist in conventional TKA, we hypothesized that the utilization of RA-TKA would likewise be lower among minority populations. Identifying patient factors that may act as barriers to access is of crucial importance, so that arthroplasty surgeons and policymakers can take steps to ensure patients of diverse backgrounds can equally experience the potential benefits of emerging technologies.

## Methods and materials

### Data source and study population

Patients were identified in the American College of Surgeons National Surgical Quality Improvement Program (NSQIP) database, which collects data from more than 700 institutions across the USA through medical chart abstraction to generate more than 150 variables on patients undergoing procedures and their outcomes during the 30 days postoperatively. Patients 18 years or older who underwent TKA from 1 January 2022 to 31 December 2022 were identified using the Current Procedural Terminology (CPT) codes 27,445 and 27,447. Only patients for whom these CPT codes appeared as their primary procedure code were included. Patients with a cancer diagnosis, a variable directly encoded into the database, were excluded. Patients with missing data on race/ethnicity or any of the below covariates were excluded. Patients were stratified by a NSQIP-encoded variable for use of a robot during the procedure.

### Variables of interest

*Outcomes of interest* The primary outcome was differences in likelihood of undergoing robot-assisted total knee arthroplasty (RA-TKA) relative to conventional, non-robot-assisted TKA based on race/ethnicity. As the NSQIP database classifies Hispanic ethnicity as a separate variable, a combined race/ethnicity variable was created where patients were classified as Hispanic if this was listed as their ethnicity, regardless of their listed race (e.g., if a patient’s race was listed as white and their ethnicity as Hispanic, they were classified as Hispanic in the new combined variable). Patients who indicated a race/ethnicity other than white, Black, Hispanic, or Asian were consolidated into a single “other” category.

*Covariates* Covariates were selected on the basis of variables that differed significantly between the TKA and RA-TKA groups on univariate analysis. Included in the final analysis as covariates were age, sex, BMI [calculated as (weight in pounds)/(height in inches)^2^ × 703], surgical setting (inpatient versus outpatient), immunosuppressant medication use, history of a bleeding disorder, preoperative infection [defined as sepsis or systemic inflammatory response syndrome (SIRS)], preoperative ascites, functional status (independent, partially dependent, or totally dependent), and American Society of Anesthesiologists (ASA) classification. Notably, these covariates have also been shown in literature to be associated with worse outcomes of TKA [18, 19].

### Patient characteristics

A total of 47,898 patients were identified who underwent TKA in 2022. Of these, 8560 (17.9%) underwent RA-TKA. Table 1 presents the characteristics of patients undergoing TKA, stratified by conventional TKA and RA-TKA. Women accounted for 60.8% of the patient population, and patients were on average 67.6 years old (range, 18–90). Age, sex, race/ethnicity, BMI, operative setting, ASA class, functional status, preoperative ascites, use of immunosuppressant medication, history of a bleeding disorder, and preoperative infection all varied significantly by whether the surgery was robot-assisted (*P* < 0.05 for all).

**Table 1:** Demographic characteristics and comorbidities of patients undergoing TKA, stratified by conventional and robot-assisted TKA (RA-TKA)

Characteristic	Total sample (%)	Conventional TKA (%)	RA-TKA (%)	*P*-value
Total	47,898	39,338 (82.1%)	8560 (17.9%)	
Age (range)	67.6 (18–90)	67.7 (18–90)	67.2 (25–90)	< 0.001
Women	29,098 (60.8%)	24,037 (61.1%)	5061 (59.1%)	0.001
Race/ethnicity				
White	36,469 (76.1%)	29,500 (75.0%)	6969 (81.4%)	< 0.001
Black	5313 (11.1%)	4593 (11.7%)	720 (8.4%)	
Hispanic	3618 (7.6%)	3101 (7.9%)	517 (6.0%)	
Asian	1383 (2.9%)	1204 (3.1%)	179 (2.1%)	
Other	1115 (2.3%)	940 (2.4%)	175 (2.0%)	
BMI (kg/m^2^) (range)	32.9 (14.8–91.6)	32.9 (14.8–91.6)	33.0 (15.7–67.0)	0.03
Outpatient setting	35,645 (74.4%)	29,175 (74.2%)	6470 (75.6%)	0.01
ASA class				
1	391 (0.8%)	307 (0.8%)	84 (1.0%)	0.03
2	21,692 (45.3%)	17,924 (45.6%)	3768 (44.0%)	
3	25,240 (52.7%)	20,638 (52.5%)	4602 (53.8%)	
4	573 (1.2%)	468 (1.2%)	105 (1.2%)	
5	2 (0.0%)	1 (0.0%)	1 (0.01%)	
Functional status				
Independent	47,401 (99.0%)	38,902 (98.9%)	8499 (99.3%)	0.004
Partially dependent	493 (1.0%)	433 (1.1%)	60 (0.7%)	
Totally dependent	4 (0.01%)	3 (0.01%)	1 (0.01%)	
History of diabetes	9259 (19.3%)	7662 (19.5%)	1597 (18.7%)	0.08
Current smoker	3191 (6.7%)	2582 (6.6%)	609 (7.1%)	0.06
History of COPD	1585 (3.3%)	1275 (3.2%)	310 (3.6%)	0.08
Ascites	6 (0.01%)	3 (0.01%)	3 (0.03%)	0.04
History of CHF	1567 (3.3%)	1313 (3.3%)	254 (3.0%)	0.08
Hypertension	31,584 (65.9%)	25,936 (65.9%)	5648 (66.0%)	0.93
Preoperative AKI	12 (0.03%)	11 (0.03%)	1 (0.01%)	0.39
On Dialysis	63 (0.1%)	55 (0.1%)	8 (0.1%)	0.28
On immunosuppressants	2062 (4.3%)	1745 (4.4%)	317 (3.7%)	0.002
Bleeding disorder	983 (2.1%)	842 (2.1%)	141 (1.7%)	0.004
Preoperative blood transfusion	13 (0.03%)	12 (0.03%)	1 (0.01%)	0.34
Preoperative infection (SIRS/sepsis)	222 (0.5%)	201 (0.5%)	21 (0.3%)	0.001

### Statistical analysis

Demographic characteristics and comorbidities were initially analyzed using univariate chi-squared analysis for categorical variables and two-tailed *t*-tests for continuous variables. A multivariable logistic regression model controlling for the above-listed covariates was then constructed to examine differences in the likelihood of undergoing RA-TKA relative to conventional TKA on the basis of race/ethnicity. The model relied on certain assumptions, including that the covariates, each of which were significant on univariate analysis, were independent and not colinear, and that there were not significant outliers influencing the model [20]. Data were analyzed using Stata statistical software: release 18; 2023 (StataCorp; College Station, TX). A *P*-value of < 0.05 was considered statistically significant.

## Results

The likelihood of undergoing RA-TKA relative to conventional TKA varied significantly by race/ethnicity (*P* < 0.001). Specifically, white patients comprised a greater percentage of RA-TKA patients relative to their percentage of conventional TKA patients (81.4% versus 75.0%), whereas Black, Hispanic, and Asian patients (and patients of any other race) comprised a lower percentage of RA-TKA patients relative to conventional TKA patients (8.4% versus 11.7% for Black patients, 6.0% versus 7.9% for Hispanic patients, 2.1% versus 3.1% for Asian patients, and 2.0% versus 2.4% for patients of any other race) (Table 1).

Table 2 presents the full results of multivariable modeling on the basis of each factor included for analysis. Relative to white patients, Black patients were 35% less likely to undergo RA-TKA (OR 0.65, 95% CI 0.60–0.70, *P* < 0.001), Hispanic patients were 30% less likely to undergo RA-TKA (OR 0.70, 95% CI 0.64–0.77, *P* < 0.001), Asian patients were 35% less likely to undergo RA-TKA (OR 0.65, 95% CI 0.55–0.76, *P* < 0.001), and patients of any other race were 22% less likely to undergo RA-TKA (OR 0.78, 95% CI 0.66–0.92, *P* = 0.003).

**Table 2:** Multivariable analysis of the impact of patient race on likelihood of undergoing robot-assisted TKA

Characteristic	Odds ratio (OR)	95% confidence interval (CI)	*P*-value
Race			
White	–	–	–
Black	0.65	0.59–0.70	< 0.001
Hispanic	0.70	0.64–0.77	< 0.001
Asian	0.65	0.55–0.76	< 0.001
Other	0.78	0.66–0.92	0.003
Age	0.99	0.989–0.995	< 0.001
Women	0.95	0.91–0.998	0.04
BMI	1.00	0.997–1.005	0.74
Outpatient	1.03	0.97–1.08	0.36
Immunosuppression	0.82	0.73–0.93	0.002
Bleeding disorder	0.76	0.63–0.91	0.003
Preoperative infection (SIRS/sepsis)	0.48	0.31–0.76	0.002
Ascites	4.48	0.89–22.38	0.07
ASA class	1.07	1.02–1.12	0.01
Functional status	0.73	0.56–0.95	0.02

Other variables that were associated with decreased likelihood of undergoing RA-TKA relative to conventional TKA included increasing age (OR 0.99, 95% CI 0.989–0.995, *P* < 0.001), female sex (OR 0.95, 95% CI 0.91–0.998, *P* = 0.04), immunosuppressant medication usage (OR 0.82, 95% CI 0.73–0.93, *P* = 0.002), history of a bleeding disorder (OR 0.76, 95% CI 0.63–0.91, *P* = 0.003), preoperative infection (OR 0.48, 95% CI 0.31–0.76, *P* = 0.002), and decreasing functional status (OR 0.73, 95% CI 0.56–0.95, *P* = 0.02). Increasing ASA class was associated with an increased likelihood of undergoing RA-TKA relative to conventional TKA (OR 1.07, 95% CI 1.02–1.12, *P* = 0.01).

## Discussion

Numerous studies have demonstrated racial/ethnic disparities in access to orthopedic procedures, particularly total joint arthroplasty (TJA) [11–13, 21]. Policies such as the Health and Human Services Action Plan to Reduce Racial and Ethnic Health Disparities have been enacted to target such disparities [22], but studies continue to demonstrate the existence of significant disparities, calling into question their efficacy in addressing access to care for minorities. The results of this study expand on the literature on racial/ethnic disparities in TJA, demonstrating that minorities have a lower likelihood of undergoing RA-TKA, an emerging technology, relative to white patients. While it is controversial as to whether RA-TKA leads to improved outcomes relative to conventional TKA, the finding that minority patients experience decreased access to RA-TKA may extend to other emerging technologies in TJA. Consequently, should these technologies prove to significantly outperform conventional TJA, this may ultimately impact outcomes for minority patients.

The finding that minority patients have reduced access to RA-TKA is in line with existing literature. Paisner et al. demonstrated that Black patients were less likely than white patients to undergo RA-TKA over the period of 2012–2020, and O’Rourke et al. demonstrated similar findings over the period of 2016–2018 [16, 17]. The results of this study expand upon these prior studies by controlling for specific comorbidities and analysis in a more recent time period, which is important given the rapidly increasing adoption of RA-TKA since 2020 [8, 9, 23–25]. Notably, per the 2023 American Joint Replacement Registry Annual Report, the use of RA-TKA more than doubled between 2020 and 2022 [9]. This underscores the importance of analysis of more recent data, as increases in utilization may exacerbate preexisting disparities, or ameliorate them as availability increases. The increase in utilization is echoed in the fact that RA-TKA comprised 17.9% of our sample, as compared with 8.6% and 6.95% in the two prior studies, respectively [16, 17]. Interestingly, no racial/ethnic disparities were seen in the utilization of RA-TKA in a study examining the earlier period of 2008–2015 [24]. This suggests that perhaps disparities have arisen in more recent years, in line with the increase in the utilization of RA-TKA, again highlighting the importance of analysis in more recent time periods. Notably, literature is inconclusive with respect to the patient-level advantages to RA-TKA. Studies seem to suggest that RA-TKA offers an advantage in improving mechanical alignment and implant position [5, 26], and may improve postoperative functional scores [5], but other studies showed no difference in the odds of early revision between RA-TKA and conventional TKA [7], as well as no significant differences in rates of complications [5, 26]. In spite of the mix of immediate and medium-term advantages and limitations posed by RA-TKA, the impact of RA-TKA on long-term outcomes remains unknown, and disparities in access to this and other novel technologies with the potential to impact outcomes remain important to investigate. Studies of disparities in other novel arthroplasty techniques have also demonstrated similar results, such as the finding that Black patients are less likely to undergo technology-assisted total hip arthroplasty [23], and that minorities are less likely to undergo unicompartmental knee arthroplasty [16, 27]. The results of this study thus augment the existing literature demonstrating an overall theme of reduced access to novel arthroplasty technologies among racial/ethnic minorities.

The causes for the disparity seen in this study are likely multifactorial. In particular, one such source is that Black patients have been shown to be more likely to undergo TKA at lower-quality, lower-volume hospitals [10, 28–30]. Several features of these lower-volume hospitals may translate into significant racial/ethnic disparities. For example, the learning curve associated with surgeon adoption of RA-TKA has been estimated to be anywhere between 6 and 20 cases as the minimum number to achieve consistent outcomes [31, 32]. Consequently, surgeons at lower-volume hospitals may not have the resources or volume to justify adoption of this technology. These lower-volume hospitals may also not have the resources to purchase robotic technologies, with the average upfront cost cited at anywhere from a few hundred thousand USD to more than one million USD, not including annual maintenance costs and disposable costs per patient [32, 33]. Hospital volume as a key driver of racial/ethnic disparities in access to RA-TKA is supported by data from Boylan et al. demonstrating that patients treated at higher-volume hospitals were more likely to undergo technology-assisted TKA [24], and Antonios et al. demonstrating a higher proportion of technology-assisted TKA occurring at urban hospitals [34]. Notably, this finding may result from the influence of cost on adoption, as noted above, or may also reflect the likely greater influence of marketing on arthroplasty surgeons at high-volume hospitals, as well as a greater eagerness or pressure to become early adopters of new technologies in academic settings [24, 25].

The fact that hospital volume is likely a significant contributor to the racial/ethnic disparity in access to RA-TKA points to cost-effectiveness as a potential area of intervention. As the average additional Medicare reimbursement for robotic assistance (Current Procedural Technology code 20,985) was 148.12 USD in 2022 [35], the higher the volume of the hospital, the more the cost is spread across more patients, and thus the higher the likelihood that the hospital system can justify the utilization of RA-TKA in its patients. This cost-effectiveness analysis was demonstrated in a recent study from Rajan et al. that showed the total cost per RA-TKA procedure decreased from 92,823 USD at low-volume hospitals to 25,730 USD at high-volume hospitals, compared with 25,113 USD for conventional TKA [33]. Importantly, it has also been noted that the cost effectiveness of RA-TKA is the highest in patients at the greatest risk for revision. Hickey et al. showed that RA-TKA can only be considered a “high-value intervention” (using the World Health Organization definition of an incremental cost-effectiveness ratio less than a nation’s gross domestic product per quality-adjusted life year) in patients at low risk for revision in a practice size of 600 at a percent utilization of 27–32%, compared with a practice size of only 100 with a percent utilization of 20% in a patient population at higher risk for revision (defined by age, BMI, and gender in that study) [36]. Thus, as minority patients have also been shown to have increased revision risk [16, 37–39], promoting equitable access to RA-TKA among minorities may be of even greater significance. Consequently, two areas in which to address racial/ethnic disparities involve increasing access to care at higher-volume hospitals for minority patients as well as reducing costs of robotic arthroplasty to potentially allow for more widespread utilization.

Although this study represents a valuable addition to the literature on disparities in access to total joint arthroplasty, several limitations must be acknowledged. First, as a retrospective study, the results of the analyses can only be interpreted in terms of association, not causation. Second, the NSQIP database is compiled by human coders, and data entry errors may exist. Furthermore, despite being conducted using a large, nationally representative database, the analyses were limited to variables collected by NSQIP. Consequently, there may have been factors that contributed to the results that were unable to be controlled for owing to to their absence from the database. For example, socioeconomic status or insurance status may have been mediating factors in the outcomes seen but are not encoded in the NSQIP database. Race/ethnicity tends to be strongly associated with various elements of socioeconomic status, though studies have shown that racial/ethnic healthcare disparities often persist at all levels of socioeconomic status [40], thus future work should aim to differentiate between the effects of race/ethnicity and socioeconomic status on access to RA-TKA. Similarly, hospital volume and geographic location may have also had an impact on the likelihood of undergoing RA-TKA. Future work should also endeavor to determine the role of these variables in the relationship between race/ethnicity and access to RA-TKA. A database also inherently cannot measure the impact of surgeon or patient preference on utilization rates of emerging technologies; thus, it is possible that these factors may have influenced disparate utilization rates of RA-TKA. With respect to surgeon preference, however, while uncertainty regarding the superiority of RA-TKA relative to conventional TKA may lead to different rates of adoption among surgeons broadly, this should not present with a racial/ethnic disparity in adoption. One additional limitation to the use of the NSQIP database is that the study inherently only examined US patients. There has been limited literature examining racial/ethnic disparities in orthopedic care abroad, and thus future studies may examine disparities in access to emerging orthopedic technologies in other geographic settings. Finally, although the analysis controlled for variables that differed significantly between patients undergoing RA-TKA and conventional TKA, these and other variables not reported may represent comorbidities and demographic characteristics that vary significantly among racial/ethnic groups and may have contributed to the observed disparity in utilization.

## Conclusions

The results of this study demonstrate that disparities exist on the basis of race/ethnicity in access to RA-TKA. This may reflect the fact that minority patients are more likely to receive care at lower-volume hospitals where RA-TKA may be less cost effective, or surgeons may not have adequate training with RA-TKA. Policies should thus aim to increase access to higher-volume hospitals and reduce costs associated with utilization of robotic systems to promote equitable access to RA-TKA for minority patients. Furthermore, this study underscores disparities in minority patient access to emerging technology in arthroplasty care more broadly. Future efforts should focus on improving this healthcare inequity to enable minority patient access to forthcoming technologies that may have a potential impact on patient outcomes.

## Data Availability

The datasets generated and/or analyzed during the current study are available in the American College of Surgeons National Surgical Quality Improvement Program repository, https://www.facs.org/quality-programs/data-and-registries/acs-nsqip/participant-use-data-file/

## Abbreviations

TKA: Total knee arthroplasty
RA-TKA: Robot-assisted total knee arthroplasty
TJA: Total joint arthroplasty
NSQIP: National Surgical Quality Improvement Program
BMI: Body mass index
ASA: American Society of Anesthesiologists
OR: Odds ratio
CI: Confidence interval
